# Using inpatient telehealth for family engagement: A mixed methods study of perceptions from patients, families, and care team providers

**DOI:** 10.1177/20552076241267374

**Published:** 2024-08-10

**Authors:** Jennifer L. Rosenthal, Jacob Williams, Keegan F. Bowers, Sarah C. Haynes, Lori Kennedy

**Affiliations:** 1 Department of Pediatrics, 8789 University of California, Davis, Sacramento, CA, USA; 2 Graduate School of Biomedical Sciences & Professional Studies, 6527 Drexel University, Philadelphia, PA, USA; 3 Pediatric Residency Program, Kaiser Oakland, Oakland, CA, USA; 4 Center for Nursing Science, 8789 University of California, Davis, Sacramento, CA, USA

**Keywords:** Telemedicine, patient-centered care, hospitalization, communication, implementation science

## Abstract

**Background:**

The Inpatient Telehealth Program permits family to remotely communicate with the patient and care team through secure, live video. We aimed to assess the implementation of this program for family engagement from the perspectives of patients, families, and providers.

**Methods:**

We used a convergent mixed methods design. The quantitative component was a cross-sectional analysis of surveys assessing patient, family, and provider experience. The qualitative component used thematic analysis of patient, family, and provider interviews plus survey free text responses. We performed memo-writing and coding. We developed hypotheses about relationships among categories and identified analytic themes. We used data transformation and narrative discussion to report the integrated findings.

**Results:**

Surveys from 214 individuals (33 patients, 145 family, 36 providers) were evaluated. Mean (standard deviation) experience ratings (1-poor, 5-best) were 4.0 (1.5) for patients, 4.6 (0.8) for family, and 4.0 (1.4) for providers. We received 134 free text responses and conducted 21 interviews. Three themes emerged: (1) inpatient telehealth enhanced patient and family experience through strengthened relationships and increased support; (2) inpatient telehealth enhanced patient care through improved information sharing and engagement; (3) low awareness of the program limited adoption. Quantitative and qualitative data aligned in that participants perceived inpatient telehealth to be valuable; however, surveys revealed that patients and providers have relatively lower satisfaction with the program.

**Conclusion:**

Inpatient telehealth for family engagement was perceived to improve family-centeredness of care. Future work is needed to overcome implementation challenges and to increase awareness of this resource among patients and families.

## Background

Family-centered care is a health care delivery approach that recognizes the collaborative partnership among patients, families, and health care providers. The Institute for Patient and Family-Centered Care defines core principles of family-centered care to include respect and dignity, information sharing, shared decision-making, and collaboration.^1^ Family-centered care is recognized to improve patient self-management, improved family experience, decreased rates of harmful errors, and reduced length of hospitalization.^2–4^ For these reasons, family-centered care is recognized as a health care standard by multiple healthcare societies, legislative bodies, and the National Academy of Medicine.^5,6^

Unfortunately, not all patients and families experience care delivery that is family-centered. Families with low income, public insurance, and from racial and ethnic minority backgrounds are less likely to receive family-reported family-centered care.^7^ Family members of minority races report more instances of experienced or observed discrimination.^8,9^ Also, during a hospitalization, family members of the patient who have a language preference other than English are more likely to report poor satisfaction, lack of knowledge of care plans, and poor family-centered care.^10–13^

The hospital setting presents unique challenges to experiencing family-centered care. Information sharing, engagement, and shared decision-making with family members of hospitalized patients is often limited due to barriers that prevent family from being physically present in the hospital to communicate with the care team and participate in patient care. Identified barriers include schedule conflicts, other cargiving responsibilities, living far from the hospital, lack of transportation, and high travel costs.^14,15^

Utilizing telehealth for hospitalized patients is a promising strategy to promote family-centered care. Telehealth permits family members to communicate with the patient and health care team through secure, live video. In this way, telehealth overcomes some barriers by making it easier to share information, support, and collaborate with the patient and family. Importantly, telehealth use facilitates the family-centered aspects that are most important to families: availability, accessibility, and communication.^16^ The use of telehealth cannot overcome all factors resulting in non-family-centered care experiences; however, telehealth offers families a resource that can potentially strengthen family-provider communication, trust, and collaboration.

Our hospital implemented an internal inpatient telehealth service line referred to as the “Inpatient Telehealth Program.” This hospital-wide program includes use of telehealth to virtually bring care team providers and family members to the bedside. Although the use of telehealth for hospitalized patients was rapidly adopted across the nation in the setting of the novel coronavirus (COVID-19) pandemic,^17–19^ research is needed to evaluate these clinical interventions.^20^ Furthermore, prior telehealth research has identified threats to longerm sustainability of such programs and highlighted the critical need for further research that focuses on implementation.^21^

Qualitative and mixed methods are valuable approaches to understanding implementation outcomes, including intervention feasibility, acceptability, appropriateness, adoption, and sustainability.^22^ The Medical Research Council process evaluation framework provides a structured approach to evaluate implementation and outcomes in real-world context.^23^ Medical Research Council domains include implementation, mechanisms of impact, and context. Based on existing literature on family-centered care, relevant outcomes to explore mechanisms of impact include information sharing, empowerment, collaboration, and support.^16,24–26^ We thus employed a mixed methods approach, applying the Medical Research Council framework, to evaluate the Inpatient Telehealth Program. Specifically, we aimed to assess the implementation of the Inpatient Telehealth Program for family engagement of hospitalized patients from the perspectives of patients, families, and care team providers.

## Methods

### Study design and setting

We conducted a mixed method study using a convergent design to examine the Inpatient Telehealth Program. Data were collected from December 2020 to October 2022. During this timeframe, the program had 372 family engagement encounters. The quantitative component was a cross-sectional analysis of a patient, family, and provider experience survey. The qualitative component was a qualitative descriptive study using thematic analysis of patient, family, and provider interviews plus free text responses from the surveys. We integrated the data using data transformation to convert the quantitative data into qualitative data.

This study was conducted at a 646-bed quaternary care hospital in Northern California. This hospital is the referral center for children and adults across a 33-county region covering 65,000 square miles. Telehealth services have been available at our center since 1992. Telehealth service lines have expanded over recent decades to encompass outpatient general and specialty care, urgent care, remote patient management, e-consults, external inpatient specialty care, and internal inpatient care.

The Inpatient Telehealth Program, an internal inpatient care service and one of our center's recently introduced telehealth offerings, was designed and implemented in May of 2020. This program was developed at the request of the health system during COVID-19 shelter-in-place orders as a service to simultaneously support clinical needs and patient and family needs. It was implemented across every unit in the hospital so that every patient, regardless of clinical service or location, could benefit from this resource. The decision to use the Inpatient Telehealth Program was at the discretion of the care team providers. In-person and virtual trainings were offered by the Health Information Technology Education Department to providers on every unit; most trainings were conducted for unit-based groups of nurse managers and bedside nurses.

We initially used Zoom (San Jose, CA) as the software interface for the program. We added ExtendedCare (Chicago, IL) as our preferred platform in January of 2021; we continued to support the use of Zoom as a secondary platform option during this study. Family members download Zoom or ExtendedCare onto their personal computer or smart device. The hospital care team uses Zoom or ExtendedCare on a tablet or computer with a speaker and microphone, which is mounted on a stand with wheels. Inpatient telehealth can be used 24/7. Care team providers such as bedside nurses or unit clerks inform patients and families of the opportunity to use telehealth for family engagement. Inpatient telehealth is also advertised on the health system's website with instructions and a 24/7 helpdesk number.

### Study population and data collection

#### Surveys

Survey data were collected from patients, family members, and providers from December 2020 to October 2022. This survey was designed and administered by the Information Technology Department; it was not evaluated for validity evidence prior to use in this study. Upon ending an Inpatient Telehealth Program encounter, a pop-up box invited users to rate their experience. Survey questions are provided in Supplement 1 and included: (1) Rate your experience using this visitation technology (1–5 stars), (2) Was the goal of your video visitation met? (Yes/No), (3) Would you use this technology in a hospital setting again? (Yes/No), (4) Rate your audio connection (1–5 stars), (5) Rate your video connection (1–5 stars), (6) Provide any other comments about this technology use (free text).

#### Interviews

We conducted one-on-one semi-structured interviews with patients, family members, and providers between January and October 2021. Participants had to have English proficiency and be aged 18 years and older. We recruited participants who had used inpatient telehealth for family engagement at least once within one month of recruitment. We initially used convenience sampling, whereby participants were identified by recommendation from providers and from the Inpatient Telehealth Activity Report. We subsequently used purposeful sampling and recruited participants with no experience using the Inpatient Telehealth Program to understand the reasons for not using the program and explore emerging insights regarding program reach. We also hoped to gain insights into how perceptions and experiences might vary under the different condition of no program use. For the latter third of the interviews, we used maximum variation sampling to identify participants with varied use of the Inpatient Telehealth Program and to achieve diversity among participants regarding gender, race, ethnicity, and role. Participants were recruited in-person or by email. Interviews were conducted either in-person in a private location at the hosptial or by videoconference, per participant preference. Sampling continued until we reached thematic saturation.^27^

The interviewer (J.W.) was a research assistant with qualitative research expertise; they had no prior relationship with participants prior to the study. They used a question guide that solicited participants’ general perceptions about the program, perceived benefits and harms, usage, facilitators and barriers to adoption, mechanisms of impact, and strategies for optimizing the program. Participants provided verbal informed consent and received a $50 gift card. Interviews were audio recorded, transcribed verbatim, deidentified, and reviewed for accuracy by the interviewer.

### Analysis

We analyzed the five quantitative survey items using descriptive statistics. We calculated proportions and means with standard deviations; we examined data by respondant type (patient, family, and provider). For qualitative data, we used thematic analysis^27,28^ with application of some principles from Grounded Theory, specifically constant comparison, theoretical sampling, and memo-writing.^29,30^ Our analysis team consisted of four researchers, including a medical student (K.B.), a clinical research coordinator (J.W.), a physician telehealth researcher (J.R.), and a nurse scientist (L.K.).

Qualitative data were analyzed concurrently with data collection. Data were examined iteratively using a framework analysis of concepts pertaining to the Medical Research Council process evaluation framework.^23^ Our initial codebook (Supplement 2) included the Medical Research Council domains (implementation, mechanisms of impact, context) merged with variables from existing literature on family-centered care.^16,24–26^

Three researchers (J.W., J.R., L.K.) used the initial transcript to independently perform analytic memo-writing and coding using a priori codes while identifying emergent codes with open coding. We met to discuss the relevance and definitions of the coding structure and new topics from inductive coding. Subsequently, the researchers independently memoed and coded 1–3 transcripts and then met to discuss memos and application of codes, refine dimensions of existing codes, add new codes, develop tentative concepts using constant comparisons, examine data for patterns and variations, and revise the interview guide. This process was repeated with every 1–3 transcripts.

We developed tentative hypotheses about relationships among categories, revisited prior transcripts in search for negative and qualifying evidence, and identified theoretical direction. We diagramed by drafting a causal network to note the relationships between variables. We created a participants’ role-ordered matrix to explore how the variables related to participants’ role types (i.e., patient, family, nurse, physician, and clerk). Free text responses from surveys were incorporated into the matrix. Recurrent unifying concepts and identified linkages and patterns between the categories became analytic themes. We used ATLAS.ti to organize and store coding and data analysis.^31^

This study was approved by the University of California Davis Institutional Review Board. This study had a waiver of the requirements for a signed consent form. The research only involved minimal risk. The level of involvement of the participants was at their own discretion. The beginning of each interview began with an introduction that included reviewing a script with consent elements. The interview only proceeds following verbal consent. For the quantitative data, the study used existing data that were collected for the purposes of clinical services. The risk of loss of confidentiality was minimized by deidentifying the data. The research could not alter the clinical treatment of patients, because data were analyzed after the clinical care was completed.

### Integration

**Figure 1.**
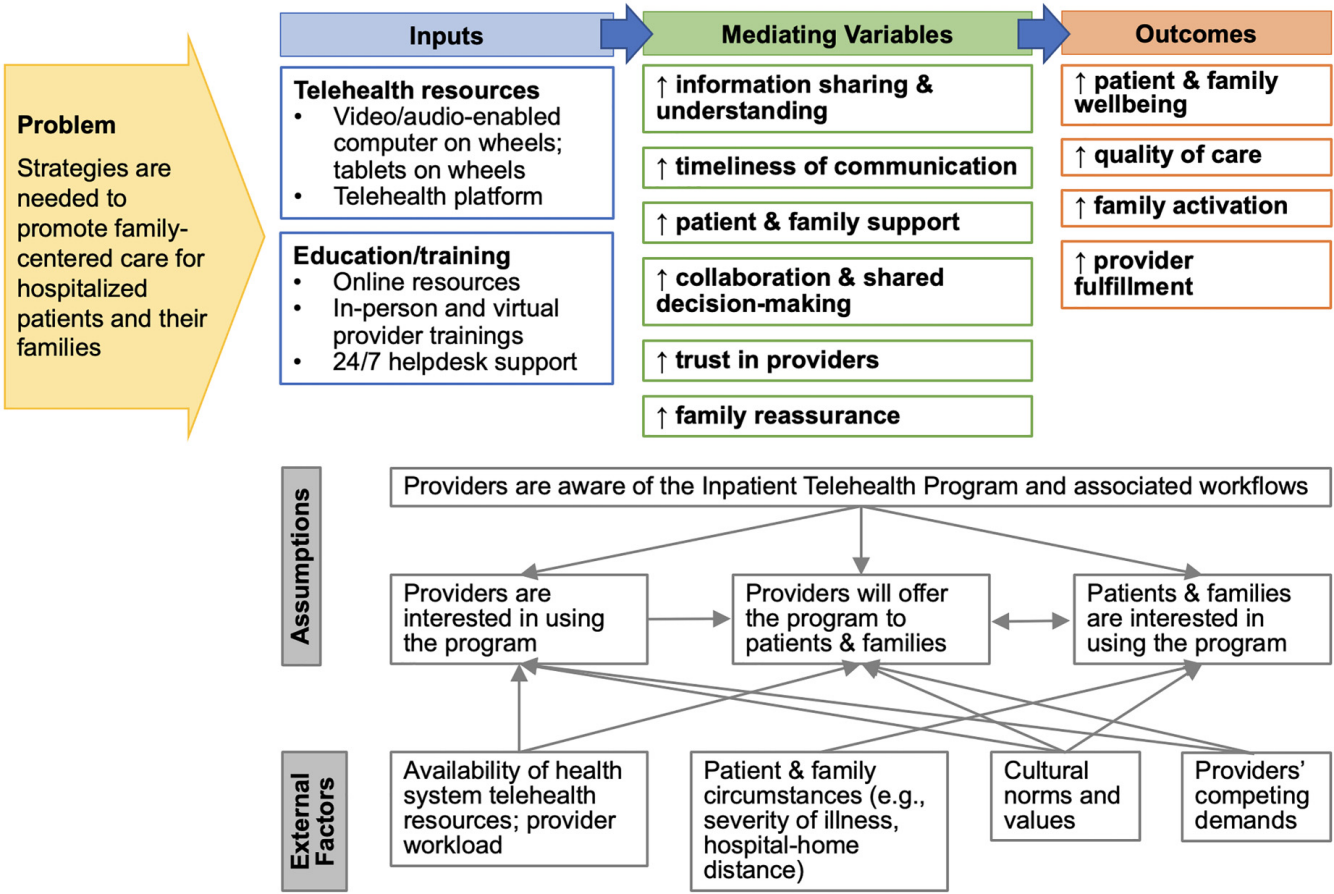
Logic model of the relationships among the inpatient telehealth program and its outcomes. *Legend*: ↑– increases or improvements. ↓ – decreases or reductions. Inputs include inpatient telehealth program components.

To compare the results of the quantitative and qualitative data, we used data transformation. We converted the quantitative data into qualitative data. Rationale for transforming in the direction of quantitative-into-qualitative data was to enrich the analyses that were informed by the Medical Research Council process evaluation framework.^23^ We identified to what extent and in what ways the two sets of results converged, diverged, or related to each other. We used a narrative discussion to report our integrated findings.^32^ We used the integrated results to create a logic model of the relationships among the Inpatient Telehealth Program's activities and outcomes (Figure 1).

## Results

Among the 372 family engagement encounters for the Inpatient Telehealth Program, 214 individuals completed the optional survey. Survey respondants consisted of 33 patients, 145 family members, and 36 providers. Survey results are presented in Table 1. The below sections present the transformed survey data and integrated results in passage format organized by theme.

**Table 1.** Inpatient telehealth user experience survey results.

Survey Item	Overall *N* = 214	Patient *n* = 33	Family *n* = 145	Provider *n* = 36
				
Rate your experience using this visitation technology,^a^ *mean (SD)*	4.4 (1.1)	4.0 (1.5)	4.6 (0.8)	4.0 (1.4)
Was the goal of your video visitation met?, *n (%)*				
Yes	187 (87.4%)	26 (78.8%)	130 (89.7%)	31 (86.1%)
No	17 (7.9%)	6 (18.2%)	8 (5.5%)	3 (8.3%)
Maybe	1 (0.5%)	1 (3.0%)	0 (0%)	0 (0%)
Would you use this technology in a hospital setting again?, *n (%)*				
Yes	192 (89.7%)	25 (75.8%)	137 (94.5%)	30 (83.3%)
No	11 (5.1%)	7 (21.2%)	3 (2.1%)	1 (2.8%)
Maybe	4 (1.9%)	0 (0%)	1 (0.7%)	3 (8.3%)
Rate your audio connection,^a^ *mean (SD)*	4.2 (1.2)	4.0 (1.4)	4.3 (1.0)	3.6 (1.6)
Rate your video connection,^a^ *mean (SD)*	4.3 (1.2)	4.4 (1.3)	4.4 (1.0)	3.9 (1.5)

SD, standard deviation.

^a^ Response option: 1 to 5 stars.

We received 134 free text responses from surveys and conducted a total of 21 interviews. We interviewed 1 patient, 4 family members, 10 nurses, 3 physicians, and 3 hospital clerks. Two of the family members had not used the Inpatient Telehealth Program; they stated that they were unaware of the program. One interview was in-person; 20 were by videoconference. Interview duration was between approximately 30 and 45 min. Table 2 shows the interview participant characteristics. Three major themes emerged from the data and are explored below. Table 3 provides exemplary quotes supporting each theme.

**Table 2.** Interview participant characteristics.

Characteristic	Patient or family *n* = 5	Care team providers *n* = 16
		
Role, *n* (%)		
Patient	1 (20.0%)	–
Family member^a^	4 (80.0%)	–
Nurse^b^	–	10 (62.5%)
Attending physician	–	1 (6.2%)
Physician resident or fellow		2 (12.5%)
Hospital clerk		3 (18.8%)
		
Age, *n* (%)		
18–39	1 (20.0%)	7 (43.8%)
40–59	2 (40.0%)	9 (56.2%)
60+	2 (40.0%)	0
		
Gender, *n* (%)		
Female	3 (60.0%)	13 (81.2%)
Male	2 (40.0%)	3 (18.8%)
Other	0	0
		
Ethnicity, *n* (%)		
Hispanic/Latinx	1 (20.0%)	1 (6.2%)
Non-Hispanic/Latinx	4 (80.0%)	15 (93.8%)
		
Race, *n* (%)		
White	3 (60.0%)	8 (50.0%)
Black	1 (20.0%)	1 (6.2%)
Asian	1 (20.0%)	5 (31.2%)
Multiracial	0	2 (12.5%)
		
Highest level of education, *n* (%)		
High school graduate	1 (20.0%)	–
Some college	2 (40.0%)	–
2-Year college degree	1 (20.0%)	–
4-Year college degree	1 (20.0%)	–
Masters or professional degree	0	–
		
Years of experience, *n* (%)^c^		
5 or less	–	5 (31.2%)
6–10	–	3 (18.8%)
11–20	–	3 (18.8%)
20 or more	–	5 (31.2%)
		
Number of times used telehealth, *n* (%)		
0	2 (40.0%)	0
1–5	0	2 (12.5%)
6–19	3 (60.0%)	3 (18.8%)
20–49	0	5 (31.2%)
50–99	0	1 (6.2%)
100 or more	0	5 (31.2%)
		
Frequency of use, *n* (%)^d^		
Never	2 (40.0%)	0
Almost never	0	2 (12.5%)
Monthly	1 (20.0%)	1 (6.2%)
Weekly	2 (40.0%)	9 (56.2%)
Daily		4 (25.0%)
		
Level of comfort using telehealth, *n* (%)		
Very uncomfortable	0	0
Slightly uncomfortable	0	1 (6.2%)
Neutral	1 (20.0%)	0
Slightly comfortable	1 (20.0%)	2 (12.5%)
Very comfortable	3 (60.0%)	13 (81.2%)
		
Type of internet access, *n* (%)^e^		
None	0	–
Dial-up	0	–
Cellular data only	0	–
High-speed or broadband	5 (100%)	–

^a^ Family members consisted of a wife, a father, and two sisters.

^b^ Nurses represented the following clinical roles: neonatal intensive care unit, adult acute care unit, and adult intensive care unit. Four of the nurses were nurse managers; one was a charge nurse.

^c^ Years of experience since completed training.

^d^ For patients and family members, frequency refers to during the hospitalization. For care team providers, frequency refers to current use.

^e^ Type of internet access for patients refers to what they have access to when not in the hospital.

**Table 3.** Extemporary quotes by theme.

**Theme 1: Inpatient telehealth enhanced the patient and family experience through strengthened relationships and increased support**	
**Sub-theme**	**Quote**
Telehealth helped to humanize the patient for the care team providers	“I always think it's important for people at the hospital to know that that patient has support by family or friends or whoever. I think even when the nurses see me—now, I’ve become a person to them instead of just another voice, and I think it helps my sister become a person to them instead of just another patient.”—Sister (013)
	“One patient, his brother and sister would call and, [telehealth] just humanizes the patient, especially because we don’t know their personality. Most of the time they’re intubated, they’re sedated.”—ICU Nurse (005)
Telehealth strengthened families’ trust in the providers	“It's always been nice to be able to see a nurse's face or a doctor's face… It becomes more of a human aspect instead of just hearing someone's voice. They’re a human being, all of a sudden… I really liked the way they spoke to my sister. I really liked the way they communicated with her and respected her… Being able to actually, physically see the nurse and her body language and everything else and how [the nurse] treated my sister, it really gave me peace of heart.”—Sister (013)
	“They see the nurses going in and out and delivering the care, they see them talking to their loved one… Bearing witness to the bathing, and the mouth washing, and the turning, and starting the medications, and all the other things that you normally would not be seeing… that also improves the layer of trust… delivering the type of care that they feel their loved ones deserve.”—ICU Nurse Manager (021)
Telehealth helped to address the needs and wellbeing of the family members	“I’ve had several patients and family members that are all over the country… We had a big [telehealth] meeting with, like, 20 family members or so in Mexico. They were never going to be able to get up to see the patient, so for them, it was a wonderful experience, because they got to say goodbye on video.”—Physician (011)
	"We just recently had one where a patient was passing and the daughter couldn’t get here from Southern California until the weekend. If she hadn’t been able to use telehealth she never would have been able to have at least some sort of opportunity to see her father and say goodbye before he passed. And he did end up passing before her flight.”—Hospital Clerk (017)
	“It does feel like we the family are also being taken care of and included… It's almost like going to visit in person, really. So, when we can see her, it really lowers our stress… It's just every time I’m able to see my sister and know that she's doing okay… It's such a positive tool to be able to use, I’m just really thankful for it.”—Sister (013)
Patients were no longer isolated from their lives outside the hospital	“Well, you’re very isolated, and it helps a great deal to be able to contact [family]. My little daughter's very pregnant right now… I get to watch my grandson walk.”—Patient (001)
	“We even had situations where we just set the iPad in front of the patient and his wife had hers on 24/7. The kids came and said goodnight to their dad every night and he got to sort of be there with the family as they were going on through the day, and that would never have been able to be possible.”—ICU Nurse Manager (021)
	“You know, we had one where she had a lot of children, a lot of children, and they all decided that they were all going to Zoom in and there were, like, six children that would every day agree that they were all going to Zoom in and be with their mother… Like, that would have never happened. We would not allow eight people to come into her ICU—with or without COVID.”—ICU Nurse Manager (021)
Increased support from telehealth facilitated patients’ healing	“You know, I was pretty bad. I’ve been here a long time. And some pretty close calls… For a while, I didn’t give a crap… It was the words of encouragement [via telehealth] that really helped… I think if someone's really depressed, you should just put [telehealth] in front of them. Give them the option at the moment.”—Patient (001)
	“I think it helps boost their spirits and their emotions. I mean, they’re essentially laying in a room with a bunch of strangers taking care of them, and no one that they love or that they’re familiar with is able to see them, so it's certainly good for them to be able to at least see their family members… I had a patient that was on a trach, was not able to talk… The family would call in, usually about once a day, to talk to the patient and check in with the nursing staff… It seemed like it gave him hope and lifted his spirits.”—ICU Nurse Manager (009)

**Theme 2: Inpatient telehealth enhanced patient care through improved information sharing and engagement**	
**Sub-theme**	**Quote**
Telehealth provided direct communication lines between providers and family members	“So now [with telehealth] you have input from literally everybody that's involved in the care. And then the family then feels like they can make an informed decision because they’re not hearing it from the pulmonologist saying, ‘Well, the nephrologist told me this and the infectious disease told me this.’ The infectious disease physician and the nephrologist are right there, and then the family can ask them very poignant questions. So that definitely improves care.”—ICU Nurse Manager (021)
	“Trying to understand what the patient is trying to tell us that we’re not understanding so we can help take care of them better and find out how we can better help them… It's happened with multiple patients recently where we’ve had some challenges… and family was able to help us figure out what was specifically going on with the patient… we’ve been able to have doctors present in the room as well as nursing staff and have a better understanding.”—Charge Nurse (007)
	“I think it's absolutely beneficial in terms of getting additional history that we may not be able to get from patients if their caregivers are the ones who dispense their medications and know more of their medical history. I think it's absolutely beneficial and achieves some of the things that we can’t obtain over a phone conversation, perhaps, in terms of they can show us their medications, what their living situation is like…”—Physician (012)
Telehealth helped to connect family members during pivotal clinical circumstances	“We’ve had multiple conversations where we’ve been able to probably transition patients more to a less aggressive disease-directed therapies and really more focus on their comfort, and make those decisions a little bit earlier… I think that [telehealth] has allowed us to have meaningful conversations with some patients and families a little bit quicker… We’ve been able to get a lot of family members on the call at the same time. And it's been a lot easier to do [telehealth] and coordinate with families, to get them all together, than it was to get them all into the hospital before.”—Physician (011)
	“So, I set [telehealth] up at 10:00 at night. Everyone was there. Everyone made a decision, and they withdrew care at that night, because they didn’t want to prolong her suffering, their mom's suffering… They were able to see their mom. So, at 10:00, everyone made a decision; we withdrew care, instead of 12:00 the next day.”—Nurse Manager (010)
	"I’ve encountered several instances where family members are out-of-state, out-of-town, or significantly long distances away where they can’t be in the hospital for family meetings or urgent situations. It's been good for those people to be able to be connected.”—ICU Nurse Manager (018)
Family members gained increased understanding of the clinical status and care plan with telehealth	“[A patient] needed to have some procedures done, and so they were able to do the informed consent on the video conferencing. Most of the time they’ve done it just by phone, but I think, again, by being able to have the family see the doctor, and the doctor see the family, and you can read facial expressions, and you can tell whether somebody really understands or doesn’t understand, or what their level of comprehension is.”—ICU Nurse Manager (009)
	“My sister had a certain vacuum on her stomach. We were able to go through step by step of actually what was happening because I didn’t understand what they were talking about over the phone… [Telehealth] was better than the phone. The nurse is able to come in with our conversation and speak to me about exactly what was happening that I could see because I had no idea where the vacuum was, and I was shocked when I saw it… It really did make a big difference from what I heard on the phone.”—Sister (013)
	“I think it's a more beneficial way to share information with patient's families, as opposed to via phone call, because you can—on video—show the patient's family. I think especially when it comes to difficult decisions or decision making with regards to end-of-life care and “Do Not Resuscitate” efforts, for the patient's family to visually see the condition of their family member is much different over video as opposed to describing it over the phone… It's allowed us to better engage with family and collaborate with them. Making informed decisions.”—Physician (012)
Increased family engagement facilitated increased family activation	“Families feel more comfortable, whether it be doing wound care at home or providing specific care related to whatever is going on with them. It's a good way for them to ask questions of the nurse at the bedside and explain to them maybe even while they’re doing a dressing change or go over specific medications and those types of things. They’re able to ask questions real-time with the nurse at the bedside with the patient… have a better understanding of what actually is going on with the patient.”—Charge Nurse (007)
	“I know that typically moms have difficulty with motivating with pumping, especially if they’re not able to be in the room and be with their baby or even see their baby. I think seeing their baby on camera has helped them focus on completing that task and being consistent… Some of them have reported they’ve been able to produce breastmilk a little easier since they’ve been able to see their child.”—NICU Nurse (008)
	“One set of triplets we had a couple months ago, the family was out of town, and they had difficulty getting transportation… The dad would call every day and ask to see his babies… I noticed a change in involvement and an uplifting in morale…. It was a CPS-involved case. I think having the video and the camera set up like that, it changed their involvement level… It kind of gave them a view of their children to give them a reason to be more involved and to want to be involved.”—NICU Nurse (008)

**Theme 3: Low awareness of the program limited adoption of inpatient telehealth**	
**Sub-theme**	**Quote**
Limited adoption was mostly due to limited awareness of the Inpatient Telehealth Program	“A lot of family members don’t realize that we have the capability at least to do like a video conferencing or telehealth.”—ICU Nurse (005)
	“My husband was in there for COVID, and he was in there for a month and one day, and for the first couple weeks because he was intubated in ICU and everything, I wasn’t able to communicate with him, but then once they removed the tube and stuff… he goes, ‘Here, let me do a Zoom call.’ I said, ‘We can do that?’ He said, ‘Yes.’”—Wife (014)
	“I think from a clinician's standpoint… More widespread information that these resources are available… Anywhere from emergency room to the ICU setting and post-operative and medical surgical and telemetry, wherever it might be… I don’t believe all of them [know about the program].”—Physician (012)
Patients and families were inconsistently offered telehealth despite the potential for it to benefit all patients and families	“Either a patient or a family member asks for it, or a nurse identifies a need for family or friends to connect with the patient and realizes that ‘Oh ExtendedCare telehealth would be beneficial,’ and then the nurse offers it… I think that the majority of patients or families are pretty aware of it. If they aren’t, I’m sure they know enough to be able to ask… I think it's pretty widely known that this is basically an option now for almost anything.”—ICU Nurse Manager (018)
	“[I offer it] for patients who are not allowed to have visitors.”—ICU Nurse Manager (010)
	“It's offered when the nurse or staff member assesses that there's family that wants to visit, or family that wants to communicate with the patient, but cannot physically come. When a patient comes into the ICU, we don’t call the family member and say, ‘Hey, do you want to Zoom with the patient?’ We really just wait for family to either show up at the unit to visit or call to get information.”—ICU Nurse Manager (009)
Providers and family members want the Inpatient Telehealth Program to be offered to all patient families	“Oh, please, this will help everyone. Offer it across the board… Not everyone will need to use it, but it will make a huge difference for the families that do need it. People can heal better when they have support and connecting with family is the best way to do that.”—Hospital Clerk (020)
	“We don’t know everyone's exact situation but we do know that seeing friends and family can be so important and telehealth is a really good way to do that. I don’t think there is a limitation to which genre might need it the most, I think everyone has the ability to need it just as much as the next person… We don’t want to miss people just because we assume… [Offering telehealth] is something that we want to be in the vocabulary all the time.”—Hospital Clerk (017)
	“There were many days that I couldn’t make it in to see my brother, but if I had the video as an option, I could have checked in on him and let him hear my voice a lot more… It would have made me feel connected to my brother. I would have had access to him if something were to go bad very quickly… Everyone should be offered it… You never know who it may help.”—Sister (015)
Providers and family members had suggestions for increasing program adoption	“People just need to be told about it and shown it. I don’t think it has to be a specific person though. Like even a front desk person could show me how to do it and that would be good.”—Father (016)
	“Make sure that they have access to it… If that's discussed at the time of admission or shortly thereafter, I think that may be useful for the patient and patient's family standpoint.”—Physician (012)
	“In an ideal world, the hospital provides [the family with] the technology to do video chat or telehealth with the patient. So that we make sure we have good, high-quality technology that has good video, good audio, and a good cell or Wi-Fi connection… We need to assess what the patients have accessibility to…. They may have a way to call, but they may not have a camera. Or they may not have a good camera. Or they may not have good internet, or secure internet.”—Physician (003)
Hospital units leveraging clerical support had strong program adoption	“Our front desk [clerks] have been really good about starting that process so that families are able to get signed up quickly [to use telehealth]… Our front desk staff have been pretty good about making that part of their admission checklist, almost that they would initiate the process of signing up [telehealth] for the family members.”—NICU Nurse (008)
	“I am the one who will set up the video conferencing… I make sure they get all set up, video is on, audio is working, before I hand them over to the nurse who then takes them into the room… We’re very used to always saying, ‘This is the visitor policy, and we offer a way to video chat with your family member.’ I don’t feel like we limit it at all, and it's always offered… It's just become part of our script for everyone… It's a super straightforward program. I don’t think that it could get any easier than it is. It's great and we need to keep supporting it for our patients and their families.”—Hospital Clerk (017)

### Theme 1: inpatient telehealth enhanced the patient and family experience through strengthened relationships and increased support

Every interview participant described how telehealth use to virtually bring family to the bedside of hospitalized patients strengthened patient- and family-provider relationships. Nurses and physicians explained how the use of telehealth humanized patients by allowing providers to meet patients’ family. Nurses and family members shared how it strengthened families’ trust in providers. Family members stated how telehealth let them witness providers’ body language with the patient, which instilled trust and reassurance. Multiple nurses and one physician expressed that this increased trust facilitated their ability to communicate with patients’ families, especially for difficult topics such as end of life conversations. One physician, however, stated that a limitation of telehealth is the inability to read body language accurately, which limits the ability to express empathy and recognize family members’ emotions. Despite this concern, they thought telehealth is superior to telephone in that it allows for a “more human touch.”

Interview and survey free text data revealed that telehealth was perceived by family and providers to be a tool that supported family members’ wellbeing and addressed their unique needs. Telehealth allowed family to visit without exposure to risks such as COVID-19. Family members could visit virtually while grieving or expressing emotions privately in their own homes. Family members shared how telehealth gave them “peace of mind” and helped them feel “very cared for” and “included and… more embraced.”

The program supported the patient, too. Participants explained how telehealth made it so patients were no longer isolated from their lives outside the hospital. With telehealth, patients could participate in routines such as family dinners or bedtime rituals. One family member wrote about talking, singing, and playing the piano to calm her son. Nurses shared stories of telehealth use lifting patients’ spirits. Similarly, a patient explained how he was depressed while hospitalized, and telehealth family visits helped encourage him to keep fighting.

While interviewees of all role types expressed that the Inpatient Telehealth Program enhanced the patient and family experience, survey data suggested that some patients did not value this program. Although almost every family member repondant reported that they would use the program again (94.5%), a smaller propoprtion of patients felt similarly (75.8%). Family member survey repsondants also had higher overall experience ratings than both the patients and providers.

### Theme 2: inpatient telehealth enhanced patient care through improved information sharing and engagement

Inpatient telehealth was perceived to enhance patient care by providing direct communication lines between providers and family. Nurses explained how telehealth family visits created dedicated time for nurses to update families about the patient's status and care plan. Direct lines of communication helped families make more informed decisions. It also was perceived by providers to improve their ability to gather relevant information. For example, providers could obtain information from telehealth connections about the patient's medical history; family members could show the providers the patient's living situation or home medications.

Providers perceived telehealth to be a tool that connected family members during pivotal clinical circumstances. It enabled large groups of family to gather quickly to discuss and to hear information. The virtual gatherings were easier to coordinate compared with in person gatherings which allowed decision-making to occur sooner. Care decisions that would typically be delayed until family was physically at the bedside were made in real-time via telehealth. This was beneficial, for example, for patients whose clinical status was rapidly declining.

Telehealth helped family members gain increased understanding of the patient's clinical status and care plan. Providers could see family members’ nonverbal cues and thus improve their ability to interpret whether the family member was adequately comprehending the conversation. Family members shared feeling more confident in their ability to make informed care decisions and relay information to other family members. Family members gained improved knowledge, skill, and confidence to care for the patient. This increased activation was perceived to benefit the patient, particularly for the transition from hospital to home. For example, family members received virtual training on discharge instructions such as wound care plans.

This perception that inpatient telehealth enhanced patient care aligns with the survey data in that most provider survey respondants reported that the goal of the telehealth encounter was met (86.1%). However, some providers were dissatisfied with the program, as only 83.3% of respondants reported that they would use inpatient telehealth again. The free text repsonses from providers suggested that the platform is a source of discontent. Providers wrote about poor audio quality, poor video quality, and delays. Also, compared to patients and family, providers reported worse ratings for audio and video quality.

### Theme 3: low awareness of the program limited adoption of inpatient telehealth

Multiple providers explained that widespread adoption of inpatient telehealth was hindered by limited awareness of the program. They shared how most patients and families did not know about this resource, but that the program's “untapped potential” must be addressed. One factor contributing to limited awareness of the program among patients and families was that providers inconsistently offered telehealth family visitations. Rather than offering telehealth use to all patient families, providers shared that the program was mostly offered to families of patients with no visitors.

Restricted offering of telehealth was possibly due to the burdens that the program placed on some nurses. Nurses stated that setting up the equipment impeded their workflow at times. Other acceptability issues among some nurses included patient privacy concerns given the inability to confirm all the individuals listening to and seeing the patient. Despite these unanticipated consequences of the program, every interviewed provider participant shared overwhelming desire for the program to continue.

Family interviewees suggested strategies to increase Inpatient Telehealth Program adoption. They advocated for targeted efforts to expand awareness, such as a marketing campaign that broadly advertised the benefits of telehealth family visitations. Multiple participants emphasized that it should be offered to all families at the time of admission. They recommended integrating it into conversations around hospital visitation policies. Some family members and providers articulated the need for additional program resources so that telehealth family visitations could be equitably accessed by all families, including those lacking broadband and smart devices. Another suggestion was to have clerical support for the program to promote expansion while minimizing burdens imposed on nurses. Although uncommon, some participants shared that the clerks in their hospital unit had the responsibility of teaching families how to use the telehealth platform, coordinating the timing of the visits with families, and setting up the telehealth equipment at the bedside. Nurses from these units expressed appreciation for clerical support, and clerks expressed their gratification in supporting the program.

## Discussion

This mixed methods study identified patient, family, and provider perspectives of using telehealth in the hospital for family engagement. Inpatient telehealth was thought to strengthen relationships, increased support, and improve information sharing and engagement. Despite these perceived benefits, adoption of the program was restricted, largely due to limited awareness of the program among patients and families. Figure 1 presents a logic model of the relationships among the program's activities and its outcomes. As shown in the logic model, multiple external factors and assumptions influence the use and impact of the Inpatient Telehealth Program. Lessons learned from this study can be used to standardize implementation and optimize the program.

Our finding that telehealth use was perceived to improve patient and family experience is consistent with prior research. A randomized controlled trial identified that the use of telehealth to virtually bring family members to the bedside during family-centered rounds improved parent-reported Child Hospital Consumer Assessment of Healthcare Providers and Systems Survey scores.^33^ Similarly, a non-randomized study reported improved family-provider communication with the use of telehealth to virtually transmit family to the bedside,^34^ which aligns with our present study's finding that telehealth improved patient- and family-provider relationships and information sharing.

Other prior research has examined unintended consequences of telehealth projects, such as the ten-project evaluation by Alami et al.^35^ Some findings aligned with our present study, including concerns that telehealth use increased providers’ workload. Regarding a result that did not align with our present study, Alami et al. found that telehealth sometimes reduced patient and family contact time with providers and made interactions less patient-centered. These results contradict our major finding that telehealth use strengthened relationships, facilitated communication with providers, and enhanced principles of patient- and family-centered care. This difference is likely explained by the way in which we used technology for our program; the focus of our use of telehealth was to support family visitations, not for direct patient care.

Another finding that diverged from prior research was that digital literacy was not a barrier expressed by our study participants. Although we did not specifically evaluate digital literacy in quantitative or qualitative data collection, we explored contextual factors influencing implementation of the Inpatient Telehealth Program. We anticipated that digital literacy might present as an influencing factor, because digital literacy is widely recognized as a significant inhibitor preventing diverse populations from using telehealth services.^36,37^ Potentially, our platform and workflow for the Inpatient Telehealth Program might be more user-friendly compared to other telehealth programs. Nevertheless, we must continue to evaluate our program reach and impact to ensure equitable access and benefit among diverse populations.

Our study is not without limitations. We limited participants to those with English proficiency. The survey used in this study has not been validated, and we do not have data on survey respondant characteristics. The qualitative phase of our study was also limited by the participation of only one patient. Although patient and family perspectives were well-represented in the survey data, inclusion of additional patient and family interviews could have potentially enriched our understanding with insights not captured in this study. Conversely, despite relatively fewer provider survey respondants, we included a comparatively larger number of provider interviewees. As such, provider perspectives about the Inpatient Telehealth Program are represented with in-depth information.

Our hospital has extensive and established support for telehealth service lines. Thus, inpatient telehealth programs at hospitals with limited resources would likely differ, limiting the transferability of our findings. We conducted our interviews during COVID-19; those results might only be applicable to this circumstance. However, survey data were collected through October 2022 when COVID-19 was a less influential contextual factor. The Inpatient Telehealth Program has continued to remain an active service across the hospital to date. Various adaptations to the program have occurred. Briefly, the Inpatient Telehealth Program now integrates with the health system's video medical interpreter platform, ExtendedCare is the only IT-supported platform for the program, and families are able to join the telehealth connection using a direct-join link without the necessity of downloading an application. Future research is needed that evaluates the current version of the program. Despite these limitations, trustworthiness of this study is supported by the use of investigator triangulation, peer debriefing (i.e., regular team meetings), and negative case analysis.

## Conclusion

This study provides valuable information on the use of telehealth for family engagement in the hospital setting, which is relevant to many hospitals aiming to establish and improve innovative family engagement solutions. The Inpatient Telehealth Program is perceived to enhance patient care and the patient and family experience. Future work is needed to identify best practices to overcome unintended consequences of provider workload burden and to enhance equitable awareness of and engagement with the program.

## Supplemental Material

sj-docx-1-dhj-10.1177_20552076241267374 - Supplemental material for Using inpatient telehealth for family engagement: A mixed methods study of perceptions from patients, families, and care team providers Supplemental material, sj-docx-1-dhj-10.1177_20552076241267374 for Using inpatient telehealth for family engagement: A mixed methods study of perceptions from patients, families, and care team providers by Jennifer L. Rosenthal, Jacob Williams, Keegan F. Bowers, Sarah C. Haynes and Lori Kennedy in DIGITAL HEALTH

sj-docx-2-dhj-10.1177_20552076241267374 - Supplemental material for Using inpatient telehealth for family engagement: A mixed methods study of perceptions from patients, families, and care team providers Supplemental material, sj-docx-2-dhj-10.1177_20552076241267374 for Using inpatient telehealth for family engagement: A mixed methods study of perceptions from patients, families, and care team providers by Jennifer L. Rosenthal, Jacob Williams, Keegan F. Bowers, Sarah C. Haynes and Lori Kennedy in DIGITAL HEALTH

sj-pdf-3-dhj-10.1177_20552076241267374 - Supplemental material for Using inpatient telehealth for family engagement: A mixed methods study of perceptions from patients, families, and care team providers Supplemental material, sj-pdf-3-dhj-10.1177_20552076241267374 for Using inpatient telehealth for family engagement: A mixed methods study of perceptions from patients, families, and care team providers by Jennifer L. Rosenthal, Jacob Williams, Keegan F. Bowers, Sarah C. Haynes and Lori Kennedy in DIGITAL HEALTH
