# Supplementary material for: Using inpatient telehealth for family engagement: A mixed methods study of perceptions from patients, families, and care team providers
Source: Digit Health. 2024 Aug 10;10:20552076241267374. doi: 10.1177/20552076241267374 (PMC11316967; doi:10.1177/20552076241267374)
Supplement: sj-docx-1-dhj-10.1177_20552076241267374 - Supplemental material for Using inpatient telehealth for family engagement: A mixed methods study of perceptions from patients, families, and care team providers [file sj-docx-1-dhj-10.1177_20552076241267374.docx]

**Supplement 1**: Survey Questions

1. Rate your experience using this visitation technology (1-5 stars)
2. Was the goal of your video visitation met? (Yes/No)
3. Would you use this technology in a hospital setting again? (Yes/No)
4. Rate your audio connection (1-5 stars)
5. Rate your video connection (1-5 stars)
6. Provide any other comments about this technology use (free text)
