# Supplementary material for: Using inpatient telehealth for family engagement: A mixed methods study of perceptions from patients, families, and care team providers
Source: Digit Health. 2024 Aug 10;10:20552076241267374. doi: 10.1177/20552076241267374 (PMC11316967; doi:10.1177/20552076241267374)
Supplement: sj-docx-2-dhj-10.1177_20552076241267374 - Supplemental material for Using inpatient telehealth for family engagement: A mixed methods study of perceptions from patients, families, and care team providers [file sj-docx-2-dhj-10.1177_20552076241267374.docx]

**Supplement 2**: Codebook

| **Code (shortened)** | **Definition** | **When to use** | **When not to use** |
| --- | --- | --- | --- |
| Contextual factors facilitating implementation of IP TH  (**context_facilitators**) | Any evidence supporting the contextual factors that facilitate being able to use IP TH | When an individual states factors that make it easy to use IP TH | When an individual states the benefits of IP TH |
| Contextual factors as barriers to implementation of IP TH  (**context_barriers**) | Any evidence supporting the contextual factors that are barriers to being able to use IP TH | When an individual states factors that make it hard to use IP TH | When an individual states the harms or uselessness of IP TH |
| Contextual factors influencing impact of IP TH  (**context_impact**) | Any evidence suggesting contextual factors that are influencing how the use of the IP TH helps [or doesn’t help] the patients/families/providers | When an individual states factors that make the IP TH program especially beneficial or especially not useful |  |
| Implementation of IP TH (**implementation**) | Any evidence supporting the process by which IP TH gets used | When an individual states how one decides whether or not to use TH, or how that process actually happens | When an individual discusses facilitators or barriers to using IP TH (which are coded as context_facilitators and context_barriers) |
| Mechanisms of impact: information sharing  (**mechanisms_information sharing**) | Any evidence suggesting how the use of the IP TH helps [or doesn’t help] with information sharing | When an individual states the process by which the use of IP TH improves [or doesn’t improve] information sharing (provider-provider or provider-family/patient or family-patient) | When an individual states there was good information sharing but doesn’t express that in the setting of using IP TH  When an individual states IP TH helps with information sharing but doesn’t express *how/why* it helps |
| Mechanisms of impact: patient or family empowerment  (**mechanisms_empowerment**) | Any evidence suggesting how the use of the IP TH helps [or doesn’t help] with empowering the patient or family members | When an individual states the process by which the use of IP TH improves [or doesn’t improve] patient/family empowerment | When an individual states there was good patient/family empowerment but doesn’t express that in the setting of using IP TH  When an individual states IP TH helps with empowerment but doesn’t express *how/why* it helps |
| Mechanisms of impact: collaboration  (**mechanisms_collaboration**) | Any evidence suggesting how the use of the IP TH helps [or doesn’t help] with collaboration among providers/family/patient | When an individual states the process by which the use of IP TH improves [or doesn’t improve] collaboration | When an individual states there was good collaboration but doesn’t express that in the setting of using IP TH  When an individual states IP TH helps with collaboration but doesn’t express *how/why* it helps |
| Mechanisms of impact: support (**mechanisms_support**) | Any evidence suggesting how the use of the IP TH helps [or doesn’t help] with supporting family or patient | When an individual states the process by which the use of IP TH improves [or doesn’t improve] support | When an individual states there was good support but doesn’t express that in the setting of using IP TH  When an individual states IP TH helps with support but doesn’t express *how/why* it helps |

IP TH – Inpatient Telehealth
